# A machine learning based sentient multimedia framework to increase safety at work

**DOI:** 10.1007/s11042-021-10984-z

**Published:** 2021-05-15

**Authors:** Gianluca Bonifazi, Enrico Corradini, Domenico Ursino, Luca Virgili, Emiliano Anceschi, Massimo Callisto De Donato

**Affiliations:** 1 Department of Information Engineering, Polytechnic University of Marche, Ancona, Italy; 2 Gruppo Filippetti S.p.A., Ancona, Italy

**Keywords:** Safety at work, Machine learning, Industry 4.0, Sentient multimedia systems, Internet of things, Fall detection, Decision trees

## Abstract

In the last few decades, we have witnessed an increasing focus on safety in the workplace. ICT has always played a leading role in this context. One ICT sector that is increasingly important in ensuring safety at work is the Internet of Things and, in particular, the new architectures referring to it, such as SIoT, MIoT and Sentient Multimedia Systems. All these architectures handle huge amounts of data to extract predictive and prescriptive information. For this purpose, they often make use of Machine Learning. In this paper, we propose a framework that uses both Sentient Multimedia Systems and Machine Learning to support safety in the workplace. After the general presentation of the framework, we describe its specialization to a particular case, i.e., fall detection. As for this application scenario, we describe a Machine Learning based wearable device for fall detection that we designed, built and tested. Moreover, we illustrate a safety coordination platform for monitoring the work environment, activating alarms in case of falls, and sending appropriate advices to help workers involved in falls.

## Introduction

During the last decades, we are experiencing a continuous increase of the attention to the safety and health of workers in their daily activities. This can be explained by the fact that statistics on injuries and deaths at work are far from reassuring. For example, Eurostat statistics reported 3,344,474 injuries and 3,552 deaths at work during 2017^1^. In this worrying scenario, many efforts to develop solutions and devices to protect workers during their activities have been made. Indeed, there are many objectives to achieve, such as predicting what is going to happen, warning in case of emergencies to respond promptly, controlling access to special areas, and so on. In such a scenario, Information and Communication Technology (ICT) has provided, and continues to provide, a great contribution. The Internet of Things (IoT) [54] and Machine Learning [50] are certainly two of the ICT sectors that are playing an increasing key role.

Smart objects, which are the protagonists of the IoT, extend the benefits of the Internet from humans to things, allowing them to interact with each other in ever smarter ways [4, 55]. They play a key role in increasing safety at work [44]. In fact, they can help to continuously and thoroughly monitor the situation in the workplace, immediately issuing alarms, which could lead to a reduction of accidents (think, for example, of gas leaks). Furthermore, smart objects can be included in the equipment of workers to monitor particular events and, in case of an accident, send the alarm and speed up rescue operations.

The fact that these objects are becoming increasingly smart and capable of making decisions and communicate with each other in increasingly complex ways has led researchers to propose sophisticated architectures capable of exploiting all this potential. For example, two architectures that take into account social relationships between objects are the Social Internet of Things (SIoT) [8] and the Multiple Internet of Things (MIoT) [9]. In both these cases, objects are becoming more and more social, and their behavior is becoming increasingly similar to human behavior. Another highly evolved IoT architecture is the Sentient Multimedia System [1, 14]. It is a distributed system capable of actively interacting with the environment by gathering, processing, interpreting, storing and retrieving multimedia information originated from sensors, robots, actuators and other information sources. As with SIoT and MIoT, end users are involved in the whole process, since they are called to communicate and express their feelings, evolving needs and requests to the devices.

The development of such complex IoT architectures has led to an enormous growth of data that must be processed in a very short time in order to obtain useful information. The most advanced solution to this problem is Machine Learning, which, not surprisingly, has had a spectacular growth in recent years [10, 49]. Machine Learning provides supervised and unsupervised learning algorithms that aim at extracting knowledge patterns from data. The knowledge extracted can be descriptive, diagnostic, but, above all, predictive and prescriptive [28]. For example, a Machine Learning based approach could analyze data from sensors installed in a working environment (e.g., data about a gas leak in a certain area) to predict a possible imminent accident (e.g., a fire) and to prescribe certain actions (e.g., alerting all personnel in that area to proceed with an immediate evacuation).

The two technologies mentioned above, i.e. Sentient Multimedia Systems and Machine Learning, represent the foundations of the framework for safety in the workplace that we propose in this paper. This framework consists of three distinct levels, namely: *(i)* *Personal Devices*, which are smart objects worn by workers (e.g., safety glasses, protective gloves, etc.); *(ii)* *Area Devices*, which are fixed smart objects associated with a specific area (e.g., access control gates, devices for controlling environmental parameters); *(iii)* *Safety Coordination Platform*, which monitors the safety of the working environment and, if necessary, activates the appropriate alarms and provides the related advices.

The design of the framework proposed in this paper is done at an abstraction level that allows it to be used in any working context and to address any safety issue. However, in order to give a very concrete idea of how it could operate in a real context, we also illustrate its specialization to a particular scenario, very studied in past literature, which is fall detection.

In fact, some of the main causes of injuries and deaths in the workplace are slips, trips and falls. Our framework adopts a new, very advanced wearable device, based on Machine Learning, which we designed, built and tested and that we describe in detail in this paper. Instead, it employs existing smart objects for Area Devices. Finally, it adopts an appropriate chain of Machine Learning based modules for the management of the Safety Coordination Platform.

We would like to point out that a detailed description of a personal device for fall detection has been presented in an our recent paper [6]. This description is taken up and partly expanded in Section 4.1 of this paper. Instead, the definition of the framework, as well as the description of its specialization to fall detection, as far as Area Devices and Safety Coordination Platform are concerned, are specific to this paper.

The outline of this paper is as follows: In Section 2, we examine related literature. In Section 3, we describe our framework in detail. In Section 4, we illustrate its specialization to the fall detection scenario. Finally, in Section 5, we draw our conclusions and have a look at possible future developments.

## Related literature

Only in the United States, thousands of deaths and disabilities occur every year because of occupational accidents [22]. Given these statistics, researchers have devoted much effort to study safety at work. For instance, in [45], the authors examine the effects of general organizational climate on safety climate and performance. As expected, general organizational climate exerts a significant impact on safety climate. In turn, safety climate is related to self-reports of compliance with safety regulations and procedures, as well as to participation in safety-related activities within the workplace. In [31], the authors propose a framework to measure perceptions of safety at work. This framework distinguishes the perceptions of the work environment from the ones of performance related to safety, and provides a systematic method for a further investigation of the impact of employee awareness, employee behavior, and organizational safety outcomes.

In addition to theoretical studies of safety at work, some researchers have focused on the practical implementation of the rules and regulations using Sentient Multimedia Models and Systems. An interesting implementation of the emergency plan, proposed in [15], exploits a multimedia approach that turns the escape plan into a multimedia software system, which integrates text, audio, video, 3D models and animations for handling emergencies in underground metropolitan transportation. The same method can be applied to a workplace context and helps workers to evacuate the building in a secure way. In [7], the authors propose a system to monitor worker movements on a construction site by collecting their raw spatio-temporal trajectory data and enriching it with relevant semantic information. The ultimate goal is the reduction of unsafe movements that may cause falls from heights, transportation accidents, exposure to harmful environments, and striking against or being struck by the moving equipment. This system helps in extracting trajectory characteristics and generates semantic trajectories to enable the desired semantic insights to better understand the underlying meaningful worker movements using the contextual data related to the building environment.

As in many other aspects of everyday life, Machine Learning has started to play an important role in safety at work. For instance, in [42], the authors propose a methodology based on Machine Learning techniques, like Classification Trees (CTs), Support Vector Machines (SVMs), Extreme Learning Machines (ELMs) and Bayesian Networks (BNs) for the analysis of the causes and types of workplace accidents. The aim of this research is the construction of an expert system with the ultimate goal of providing a tool that facilitates the elaboration of a workplace accident prevention policy. In [59], the authors apply two Machine Learning models, i.e., Random Forest (RF) and Stochastic Gradient Tree Boosting (SGTB), to a dataset extracted from a large pool of textual reports on construction injury via a highly accurate Natural Language Processing (NLP). The ultimate goal is the prediction of injury type, energy type, and body part with a high accuracy.

In this paper we specialize our framework description to fall detection, so it is interesting to evaluate the approaches addressing this issue in literature. Preliminarily, we note that most of these approaches are focused on elderly people, while ours is conceived for a work environment. In this context, an operator can perform activities that can be easily confused with falls, like running and jumping. Wearable devices for fall detection are usually made with accelerometers and gyroscopes, which can easily confuse sport activities with falls. For this reason, we used the Daily and Sports Activities Dataset (see Section 4.1.1) to train our classification algorithm, in order to make it more resilient to different types of activities.

There are three different types of techniques developed for fall detection, namely ambient sensor based, vision based, and wearable device based [43].

Ambient sensor based techniques exploit the recordings of audio and video and/or monitor vibrational data from the environment [19, 21, 57, 60, 62]. For instance, the approach described in [57] analyzes and verifies sensor-transmitted events through audio and video streams for object detection and tracking. This approach is based on a wireless badge node between the user and her/his network. It detects falls through an event sensing function and a continuous tracking of the approximate location of the user. Another interesting approach is the one proposed in [62]. Differently from the previous approach, it uses the audio signal from a single far-field microphone. Classification is obtained using a Support Vector Machine with a Gaussian Mixture Model. This way, each fall is modeled as a noise segment. Other kinds of ambient sensor have been used in the past, like pressure sensors in [3], or a floor sensor based on near-field imaging in [51]. Both of them consider the movement and location of objects on the floor to classify people’s actions. Approaches based on ambient sensors are not intrusive, but they are costly and difficult to install, because it is necessary to setup the whole room with sensors.

The second category of fall detection approaches comprises those based on vision [24, 25, 27, 40, 46]. For instance, the approach described in [25] uses an artificial vision algorithm to detect the person with a camera and to study changes in human actions. A Machine Learning algorithm classifies the current state of the user. In [46], the authors propose a framework for indoor scenarios using a single-camera system. When this system detects a very large motion, with a direction less than 180^∘^, it continues to monitor the next 50 frames. If some conditions are verified, a fall might have happened, so it monitors 25 more frames. If no other movements, or just a small one, occur, then that motion is considered a fall, and a warning signal is sent out. Vision based approaches can be really accurate, but they need cameras in all rooms to monitor, causing a high installation cost.

The last category of fall detection approaches relies on wearable devices [5, 11, 34, 36–38, 48, 53, 61]. These devices are made up of different kinds of sensor. For instance, the approach described in [35] uses different wireless tags placed on some parts of the body to detect the posture of a user. Acceleration thresholds, along with velocity profiles, are applied to detect falls. Another, less invasive, approach is the one presented in [41]. It uses waist-mounted accelerometers to detect a fall when negative acceleration suddenly increases. A similar proposal is found in [58], where the authors design a wearable airbag with an accelerometer and a gyroscope.

Other fall detection approaches are based on Machine Learning. For instance, the approach of [52] monitors tri-axial accelerometer data in three different sliding time windows, each one lasting one second. The system relies on a sensing unit, like a mobile phone. Something similar is described in [47]. In this approach, wearable sensors are placed in six different positions of the body. The authors test six different Machine Learning algorithms to find the best one. This approach obtains very satisfying accuracy values, but it could be invasive for the final user. Wearable-based approaches have several advantages because they are affordable, easy to install and use. In addition, they are independent from the environment, because sensors are placed on the body. However, the computational power of the devices hosting them is low.

## Framework description

**Fig. 1:**
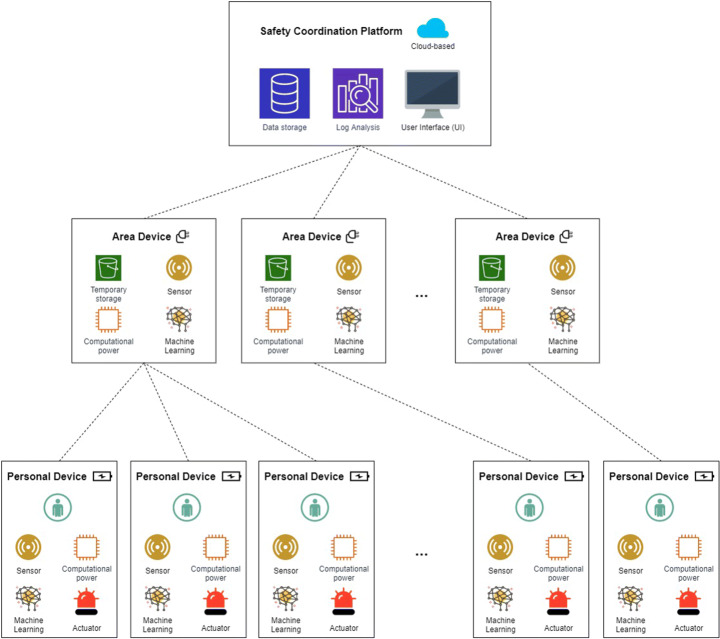
The overall architecture of the proposed framework

The overall architecture of the proposed framework is shown in Fig. 1. It assumes that the global working environment is partitioned in several areas where workers operate. These areas, along with their corresponding smart objects, are fixed. Instead, workers, with their wearable smart objects, move from one area to another over time.

As can be seen from Fig. 1, this architecture consists of three distinct layers, namely:

- *Personal Devices*: these are smart objects worn by workers. They have a twofold objective, i.e., supporting a worker in her/his work activities and guaranteeing the maximum safety at all times.
- *Area Devices*: these are fixed smart objects, each associated with a specific area. They aim at constantly monitor the area which they belong to, in order to support safety. For this purpose, they process both the data produced by themselves and the ones coming from the Personal Devices of the workers present in the area at that moment.
- *Safety Coordination Platform*: it represents the highest layer of our framework. It receives data from all the Area Devices of the working environment and is responsible for processing these data to ensure the overall safety of the whole environment.

The communication between the Area Devices and the Safety Coordination Platform is point-to-point, while the communication between Area Devices is broadcast. The same happens for the communication between Personal Devices, as well as for the one between Area Devices and Personal Devices.

In a certain area, there is only one Area Device of a given type, while there may be several Area Devices of different types. At a certain time, a Personal Device can communicate with the Area Devices of the area where it is located. As a result, it can exchange data with multiple Area Devices, each having a type different from the ones of the other Area Devices. However, given a certain type, it can communicate only with the Area Device of that type, located in the area where the operator wearing it is working at that moment.

Obviously, a Personal Device worn by the operator moves with her/him. Therefore, when she/he moves from one area to another, the Device Areas with which her/his Personal Device communicates, also change.

In the next subsections, we provide a more detailed description of the devices that make up our framework.

### Personal Devices

**Fig. 2:**
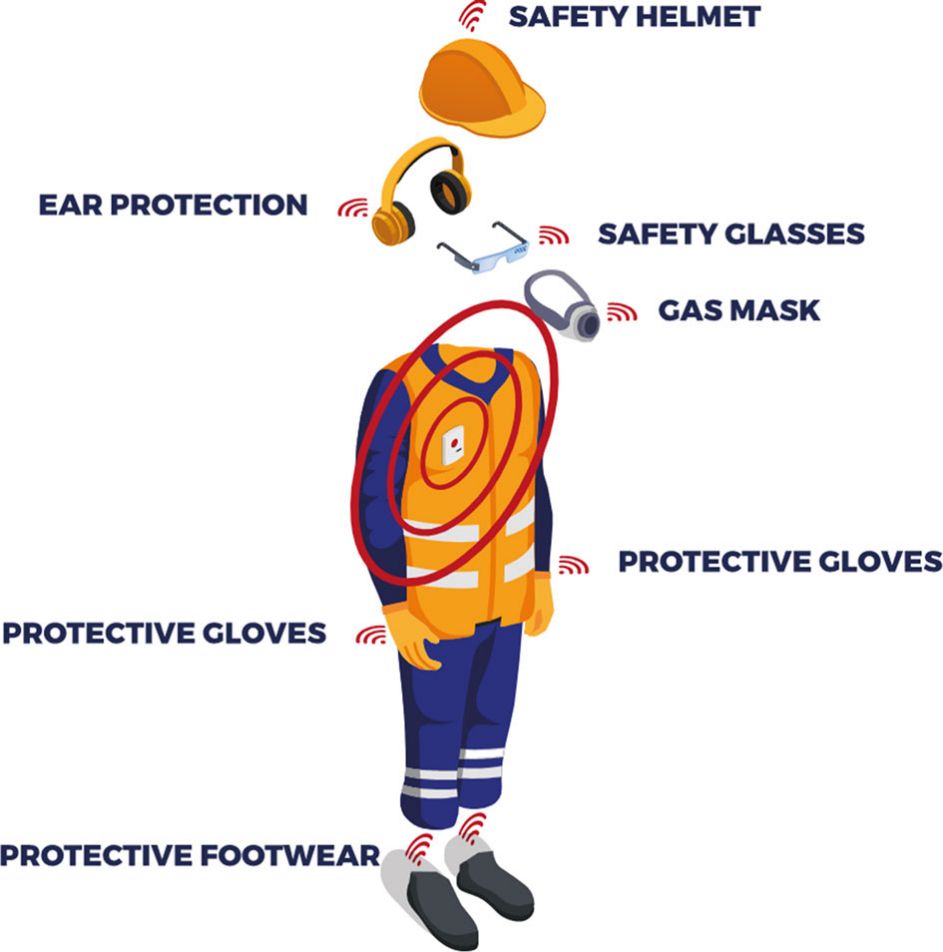
An overview of Personal Devices available for a worker

The focus of Personal Devices is the individual worker. Their goal is supporting the operator wearing them in all her/his activities, ensuring her/his safety at all time. For example, at this layer of our framework, we can find devices for augmented reality, which aim at showing the user how to perform certain operations, devices for taking the minimum path to evacuate an area or to reach an injured colleague, or devices for the detection of falls, to promptly report any injuries from tripping, slipping, etc. Fig. 2 provides an overview of these devices.

A smart object belonging to the Personal Device layer of our framework must meet some requirements. More specifically:

- It must be able to collect data that allows the derivation of information about the worker who is wearing it and the area where she/he is operating. For example, such a smart object could have accelerometric and gyroscopic sensors, to determine the motion or activity that the worker is performing, and/or a camera, to monitor her/him while she/he is performing some dangerous activities.
- It must have enough computational power to ensure an initial analysis of data retrieved by it. This feature makes this first layer of our framework an edge-computing network, and therefore a network of smart objects capable of performing real time analysis without the aid of a cloud service. Many of the data analyses performed by smart objects is based on Machine Learning. An example of how Machine Learning can be used to analyze data and make decisions within these smart objects is shown in Section 4.1.3.
- It must be able to carry out “actions” helping the operator in her/his activities or signaling a danger or, even, sending an alarm (for instance, in the event of an accident to the worker). For example, a smart object that monitors the position of the operator must be able to issue vibrations, sounds or activate appropriate LEDs, to report her/his possible entry into a restricted access area or the presence of another operator at a distance less than that required by safety regulations against COVID-19. This implies that each of these smart objects must be equipped with one or more actuators.
- It must have the ability to communicate with other smart objects, as well as with other kinds of device.
- It must be powered by a battery and it must be able to continuously monitor the corresponding charge, in order to alert the operator if a recharge is needed.
- It must have a low power consumption, avoiding as much as possible the need to recharge the battery during a work shift of its operator.
- It must be wearable and, if possible, non-invasive.

### Area Devices

The focus of Area Devices is the monitoring of a specific area in a working environment. To achieve this goal, the smart objects belonging to this category leverage both the data produced by them and the one sent by the Personal Devices present in the area. The ultimate goals of the monitoring performed by them are the prevention of accidents, the optimization of environmental parameters to improve the quality of the operators’ work, the control of access to specific reserved areas, and so forth.

An example of an Area Device is represented by the fixed smart objects for the detection of falls through video that, as we will see, can be used in parallel with Personal Devices for fall detection. A second example could be a smart object for the analysis of vibrations in structures and plants, able to detect an imminent failure of the structure or an imminent danger and to warn immediately the workers who are in the area.

A smart object belonging to the Area Device layer of our architecture must meet certain requirements. More specifically:

- It must contain some sensors that are able to measure environmental parameters, such as temperature, brightness, presence of specific gases, etc.
- It should communicate with other smart objects and with the Safety Coordination Platform through various modes and protocols, like Bluetooth, WiFi, UWB (Ultra Wide Band), etc.
- It must have a computational power allowing it to carry out real-time data analyses and to support the execution of Machine Learning and other Artificial Intelligence techniques in order to perform real-time predictions.
- It must be able to temporarily save some data in order to keep track of communications with other Area Devices and Personal Devices. For example, it must need to store that, at a certain time, there was a gas leak in the area of interest or that, in the same area, the temperature was above a certain threshold.
- It must be able to be connected to the power grid. Furthermore, it should be equipped with a backup battery in case of a power failure.

Area Devices represent the intermediate layer of the proposed framework and, therefore, play a key role in guaranteeing the communication between the other two layers, i.e., Personal Devices and Safety Coordination Platform.

### Safety Coordination Platform

The Safety Coordination Platform represents the highest layer of our framework. It aims at monitoring the situation of the whole working environment based on the data provided by the Area Devices. For this purpose, it carries out the appropriate data analysis and makes the suitable decisions regarding any alarms and/or requirements. This is possible thanks to its cloud-based nature, which allows it to be accessed at any time while ensuring an excellent level of scalability and availability.

In order to be able to perform all the activities required, the Safety Coordination Platform must be capable of saving large amounts of data in the form of logs, event alerts, structured databases storing the characteristics and positions of the devices, and so on. Some of these data must be processed continuously in real time while others must be considered only for particular knowledge extraction activities. For this reason, the Safety Coordination Platform is equipped with a data lake, appropriate algorithms for the extraction of semantic relationships between data stored in different sources (such as synonymies, homonymies, etc.), and data integration techniques, like the ones described in [16, 17, 26, 39].

Given its cloud-based nature, a possible implementation of the Safety Coordination Platform could involve Apache Kafka^2^ installed on a cloud node (or on a cluster of nodes, depending on the size of the scenario under consideration). Kafka aims at managing the data flow produced by Personal Devices and Area Devices. The Kafka Publisher saves the flow data in the data lake; the latter could be managed through Kylo^3^. Data stored in the data lake can be processed through the ELK stack^4^. In particular, Logstash can be used to extract data from the data lake, perform some cleaning operations on it and pass cleaned data as input to Elasticsearch. The latter can be used to perform the appropriate analyses on the data received from Logstash. Finally, Kibana can be adopted to create different dashboards to display the data processed by Elasticsearch, to monitor the overall workspace environment and to set alarms based on control thresholds.

Based on all available data, the Safety Coordination Platform can, first of all, carry out descriptive and diagnostic analyses [23]. Thanks to them, it can monitor and show in real time the values of a set of Key Performance Indicators (hereafter, KPIs) that describe the situation of the working environment areas. Examples of KPIs that could be adopted in this case are the number of reported accidents and incidents, the time injury frequency rate, the time injury incidence rate, the number of equipment breakdowns, and so on. Overall, KPIs regard the work performance of each area and, above all, the safety of the operators who are working within it. Monitored data are represented in a dashboard and, whenever one KPI exceeds a certain threshold, the Safety Coordination Platform activates the corresponding alerts through the appropriate mechanisms and actuators with which it is equipped.

The presence of a data lake, as well as of high and flexible computing power, allows the implementation, within the Safety Coordination Platform, of appropriate Machine Learning and Artificial Intelligence based approaches, capable of supporting predictive and prescriptive analyses. The results of these analyses give rise to appropriate knowledge patterns that can support decision makers in taking a set of actions to further improve the safety of the working environment.

For example, a thorough analysis of the logs could help to better tune the values of the actuator activation parameters in order to minimize the presence of false positives while keeping false negatives as low as possible (or, better, equal to zero). As a second example, the analysis of the various sensors of brightness, temperature, humidity, etc., relative to a year just passed, can lead to suggest a series of actions to be taken in certain areas of the working environment in order to improve the thermo-hygrometric well-being of the operators working therein.

## Specialization of the proposed framework to fall detection

In Section 3, we have provided a general description of our framework. In this section, we want to show its behavior in detail, defining and testing the suitable Machine Learning algorithms for the extraction of knowledge about safety at work from the available data. In order to provide a detailed description of both the algorithms and the experiments, we must focus on a particular case of safety at work; for this purpose, we choose fall detection. This choice is motivated by the fact that some of the main causes of accidents in workplaces all over the world are slips, trips and falls. As shown in Section 2, there are three different kinds of technique developed for fall detection, namely ambient sensor based, vision based, and wearable device based [43]. In the following, we focus on wearable device based techniques.

In the description of how our framework handles fall detection, we put a particular emphasis on the Personal Devices layer, as we have designed, built and tested an ad hoc smart object that implements Machine Learning techniques for fall detection starting from the data it derives through its sensors. Instead, as far as the Area Devices layer is concerned, we have used some fixed devices for fall detection already existing. Finally, as for the Safety Coordination Platform layer, we have defined a chain of Machine Learning based modules that uses the data provided by both the Personal Devices and the Area Devices to decide whether or not to activate an alarm and, in the affirmative case, to coordinate rescue operations.

### Personal Devices for fall detection

In this section, we describe a Personal Device for fall detection that we designed, built and tested. Since it is based on Machine Learning, we first had to build a support dataset; we illustrate it in Section 4.1.1. Then, we had to make some descriptive analyses on the available dataset in order to better understand the reference context and the problems to face; we describe them in Section 4.1.2. Starting from the results of these analyses, we could define the Machine Learning algorithms that our device could have implemented; we report them in Section 4.1.3. Once determined the best algorithms, we had to identify the most suitable hardware to implement them; we discuss this issue in Section 4.1.4. After having chosen the appropriate hardware, we had to embed our application logic on it; we discuss this activity in Section 4.1.5. Finally, once realized the device, we had to test it; we discuss our testing activity in Section 4.1.6.

#### Support dataset outline

In recent years, scientific community has highly explored wearable device based approaches for fall detection, especially due to the pervasive diffusion of portable devices, like smartphones, smartwatches, etc [18]. Many public datasets can be found online to perform analyses on slips, trips and falls, but also to define new approaches for their detection and management. We chose four datasets for our training and testing phases among all those analyzed. In particular, we selected those datasets that would help us define a generalized model, able to comply with the different activities performed by workers and operators of various sectors, who normally make very different moves during their tasks.

“SisFall: a Fall and Movement Dataset” (hereafter, SisFall) is the first dataset used. It was created by SISTEMIC, the Integrated Systems and Computational Intelligence research group of the University of Antioquia [56]. This dataset consists of 4505 files, each referring to a single activity. Activities are grouped in 49 categories; 19 of them are ADLs (Activities of Daily Living) performed by 23 adults, 15 are falls (Fall Activities) that the same adults had, and 15 are ADLs carried out by 14 participants over 62 years old. All the data were collected using a device placed on the volunteers’ hips. This device includes different kinds of accelerometer (ADXL345 and MMA8451Q) and a gyroscope (ITG3200).

“Simulated Falls and Daily Living Activities” (hereafter, SFDLAs) is the second dataset used. It was created by Ahmet Turan Özdemir of the Erciyes University and by Billur Barshan of the Bilkent University [47]. It is made up of 3,060 files regarding 36 different activities carried out by 17 volunteers. Each task was repeated about 5 times by each volunteer. Specifically, the 36 activities are 20 Fall Activities and 16 ADLs. All data was recorded using 6 positional devices placed on the head, chest, waist, right wrist, right thigh, and right ankle of each volunteer. The wearable device was made up of an accelerometer, a gyroscope and a magnetometer.

“CGU-BES Dataset for Fall and Activity of Daily Life” (hereafter, CGU-BES) is the third dataset used. It was created by the Laboratory of Biomedical Electronics and Systems of the Chang Gung University [20]. It consists of 195 files referring to 15 volunteers who performed 4 Fall Activities and 9 ADLs. All data was collected using both an accelerometer and a gyroscope.

“Daily and Sports Activities Dataset” (hereafter, DSADS) is the fourth, and last, dataset used. It was created by the Department of Electrical and Electronic Engineering of the Bilkent University [2]. It is a collection of 9,120 files regarding 152 activities carried out by 8 volunteers. Each activity, which lasted about 5 minutes, was split into 5 seconds long recordings. Contrary to the other three datasets, this regards sport activities. The reason why we chose this dataset is to make our model generalizable, more resilient to the various situations occurring in a working environment. All data were collected with the usage of 5 sensors with an accelerometer, a gyroscope and a magnetometer. Each of them was placed on different parts of the volunteer’s body.

The information we used was the one extrapolated from the accelerometers and gyroscopes. The reasons of this choice were two. The first one is data availability; indeed, acceleration and rotation were measurements found in all datasets. The second one concerns the better performances obtained by Machine Learning models than thresholding based models when using accelerometric data [30]. Acceleration and rotation data from the four datasets were merged to obtain a new dataset. It consists of a table with six columns reporting the values of acceleration and rotation along the X, Y, and Z axes. The structure of this table, together with some example tuples, is shown in Table 1. It is made up of 8,579 activities, where 4,965 are not falls while 3,614 are falls. Each file, linked to an activity, stores all the values of the 6 parameters considered for some samples.

**Table 1:** Structure and some example tuples of the merged dataset

*Acceleration*_*X*_	*Acceleration*_*Y*_	*Acceleration*_*Z*_	*Rotation*_*X*_	*Rotation*_*Y*_	*Rotation*_*Z*_
14.529	67.413	− 12.506	18,271	− 955.762	− 9.447
14.383	65.208	− 12.375	14.776	− 951.406	− 4.152
14.310	65.671	− 15.453	13.564	− 950.841	− 7.296
15.674	68.120	− 13.910	19.656	− 948.253	− 4.601
14.814	68.475	− 15.168	19.234	− 949.437	− 6.797

Obviously, as the data comes from different datasets, the number of samples for each activity is not homogeneous; indeed, it is determined by the length of the activity and the sampling frequency used in the original dataset it comes from. However, different activity lengths and sampling frequencies do not significantly affect the final result, as long as sampling frequency is much higher than the activity length. This is true in all our datasets, because our features are barely influenced by the number of samples available. This happens not only for the maximum and the minimum values, which is intuitive, but also for the mean value and the variance, because the more the number of samples increases the more both the numerator and the denominator of the corresponding formulas grow.

In order to reduce as much as possible the noise from our dataset, we applied a Butterworth Infinite Impulse Response (IIR) second order low-pass filter, with a cut-off frequency of 4 Hz to it. In this task, we kept the frequency response module as flat as possible in the passband. The Butterworth filter (also known as maximally flat magnitude filter) was chosen by us for its simplicity and low computational cost [33]. This filter was first described by Stephen Butterworth [13] in 1930. Its frequency response is maximally flat in the passband and rolls off toward zero in the stopband. These features make it perfect for our case of interest and for a future hardware implementation. In addition to using the Butterworth filter, we deleted all the excess data and made the appropriate adjustments to it by performing some Extraction, Transformation and Loading (ETL) activities. More specifically: *(i)* we removed all the rows containing null values; *(ii)* we removed all the columns not containing accelerometric and gyroscopic data; *(iii)* we replaced all the commas with decimal points; *(iv)* we trimmed all the blank values.

We then proceeded to the feature engineering phase. Specifically, we considered 4 features of a parameter *ζ*, which sampled data was in our dataset. Those 4 features are the maximum value, the minimum value, the mean value and the variance of *ζ*. Given *n* the number of samples of *ζ* in our dataset and *ζ*[*k*] the value of the *k*^*th*^ sample of *ζ*, 1 ≤ *k* ≤ *n*, the definition of the 4 features is shown in Table 2.

**Table 2:** Feature definition

Feature	Definition
Maximum Value	$\max \limits _{k=1..n}(\zeta [k])$
Minimum Value	$\min \limits _{k=1..n}(\zeta [k])$
Mean Value	$\mu =\frac {1}{n}{\sum }_{k=1}^{n} \zeta [k]$
Variance	$\sigma ^{2} = \frac {1}{n}{\sum }_{k=1}^{n}(\zeta [k] - \mu )^{2}$

As shown in Table 1, our dataset contains 6 parameters, corresponding to the values of the *X*, *Y* and *Z* axes returned by the accelerometer and the gyroscope. So that, each activity is described by 24 features in total, having 4 features for 6 parameters at disposal.

Finally, in a very straightforward way, we can label each activity with one of two classes, which possible values are *Fall Activity* and *Not Fall Activity*.

We obtained an 8,579 × 25 matrix, representing the training set used to perform the next classification activity.

#### Preliminary analyses on the support dataset

**Fig. 3:**
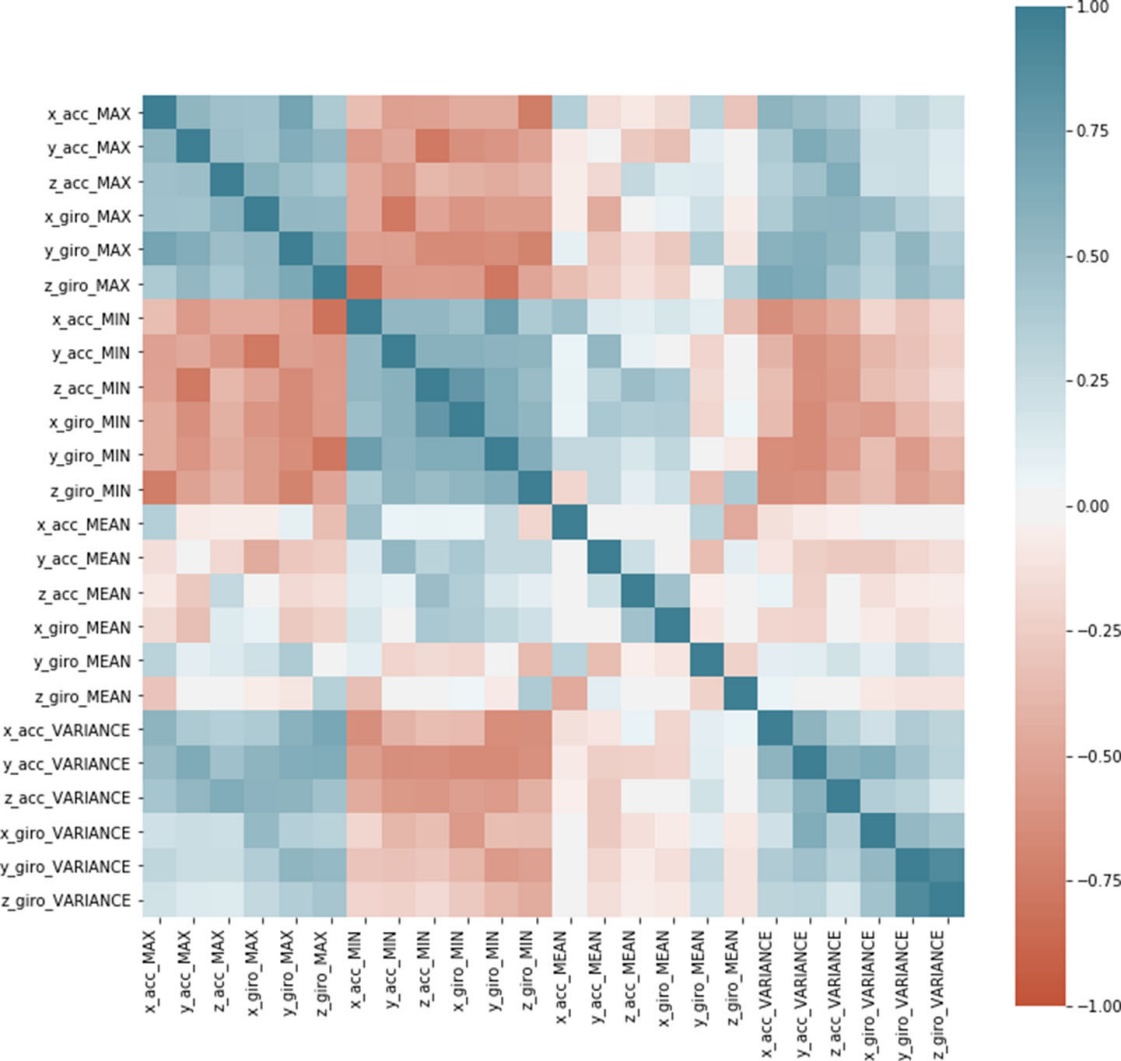
Correlation matrix between the features

In this section, we present some of the analyses done on the support dataset. They allowed us to better understand the context we were working in and to better face the next challenges. First, we computed the correlation matrix between the features. It is reported in Fig. 3.

Looking at this matrix, it is evident that some negative correlations exist between the maximum and minimum values of some parameters. In addition, we can notice a positive correlation between the maximum values (resp., minimum values, variances) computed on the various axes and on the two sensors. On the other hand, some parameters have neither positive nor negative significant correlations. A result particular evident is where the feature “mean value” is involved, because in all these cases the correlation is almost always null. This suggests that these last parameters would have been crucial in the next classification activity.

In order to verify this last intuition, we generated a list of features using a Random Forests algorithm [12] with a 10-Fold Cross Validation [32]. The features of the list were sorted according to their relevance in identifying the correct class of activities.

The algorithm used, given a decision tree $\mathcal {D}$ having *N* nodes, computes the relevance *ρ*_*i*_ of a feature as the decrease of the impurity of the nodes splitting on *f*_*i*_ weighted by the probability of reaching them [29]. The probability of reaching a node *n*_*j*_ can be computed as the ratio of the number of samples reaching *n*_*j*_ to the total number of samples. The higher *ρ*_*i*_, the more relevant *f*_*i*_ will be. Formally speaking, *ρ*_*i*_ can be computed as:

$$ \rho_{i} = \frac{{\sum}_{n_{j} \in N_{f_{i}}} \vartheta_{j}}{{\sum}_{n_{j} \in N} \vartheta_{j}}$$

Here, $N_{f_{i}}$ is the set of the nodes of *N* splitting on *f*_*i*_. *𝜗*_*j*_ is the relevance of the node *n*_*j*_. If we assume that *n*_*j*_ has only two child nodes *n*_*l*_ and *n*_*r*_, then:

$$ \vartheta_{j} = w_{j} C_{j} - w_{l} C_{l} - w_{r} C_{r} $$

Here:

- *w*_*j*_ (resp., *w*_*l*_, *w*_*r*_) is the fraction of samples reaching the node *n*_*j*_ (resp., *n*_*l*_, *n*_*r*_);
- *C*_*j*_ is the impurity value of *n*_*j*_;
- *n*_*l*_ (resp., *n*_*r*_) is the child node derived from the left (resp., right) split of *n*_*j*_.

The value of *ρ*_*i*_ can be normalized to the real interval [0,1]. To do this, it must be divided by the sum of the relevances of all features.

$$ \overline{\rho_{i}} = \frac{\rho_{i}}{{\sum}_{f_{k} \in F} \rho_{k}} $$

where *F* denotes the set of all the available features.

The final relevance of a feature *f*_*i*_ returned by Random Forests is obtained by averaging the values of the normalized relevances $\overline {\rho _{i}}$ computed on all the available trees:

$$ \widehat{\rho_{i}} = \frac{{\sum}_{t_{q} \in T} \overline{\rho_{i}}}{|T|}$$

Here, *T* is the set of all the trees returned by Random Forests.

Table 3 shows the result obtained with the approach explained above applied to the features of our interest.

**Table 3:** Feature relevance in identifying the correct class of activities

Feature	Relevance
y_acc_MEAN	0.2435
y_acc_MAX	0.1877
x_acc_MIN	0.1004
y_acc_MIN	0.0545
x_gyro_MEAN	0.0504
z_gyro_MEAN	0.0357
z_gyro_MIN	0.0336
y_gyro_VARIANCE	0.0326
y_acc_VARIANCE	0.0298
z_acc_VARIANCE	0.0293
x_acc_MAX	0.0283
x_gyro_VARIANCE	0.0269
z_acc_MIN	0.0255
z_gyro_VARIANCE	0.0221
y_gyro_MIN	0.0175
z_acc_MAX	0.0138
x_acc_MEAN	0.0127
z_gyro_MAX	0.0103
z_acc_MEAN	0.0095
x_acc_VARIANCE	0.0095
y_gyro_MAX	0.0090
x_gyro_MIN	0.0081
x_gyro_MAX	0.0052
y_gyro_MEAN	0.0041

**Fig. 4:**
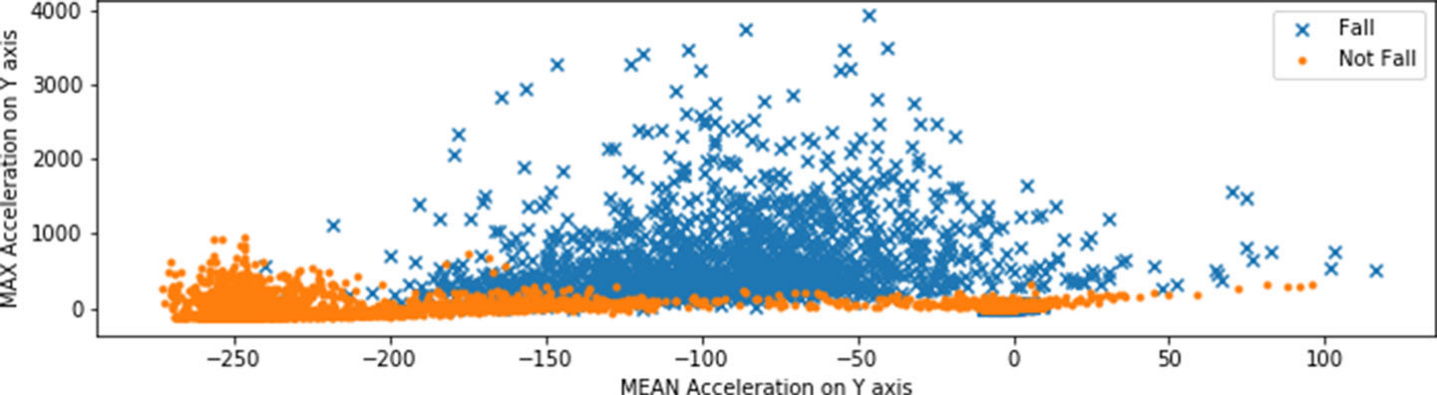
Activities labeled as *Not Fall* and *Fall* against the mean and the maximum accelerations on the *Y* axis

Then, we wanted to check if what Random Forests suggested made sense. So, we took the two features with highest relevance that this algorithm returned, i.e., the mean and the maximum accelerations computed on the *Y* axis. In Fig. 4 we show the scatter diagram drawn from these two features. Each orange dot is an activity labeled as *Not Fall*, while each blue cross is an activity labeled as *Fall*. We can see that the *Not Fall* activities has a very negative mean acceleration and a much lower maximum acceleration than the *Fall* ones. As a consequence, we can conclude that Random Forests actually returned a correct result rating these two features as the most relevant ones. Indeed, it is easy to distinguish falls from not falls with their combination.

#### Detecting a classification approach to apply on the available dataset

After having built a dataset for the training task of our Machine Learning campaign, we proceeded in our research with the definition of the classification approach to be natively implemented in the Machine Learning Core of LSM6DSOX, i.e., the sensor at the base of our device. Firstly, we verified if one (or more) of the existing classification algorithms, already proposed, tested, verified and accepted by the scientific community, obtained satisfactory results in our specific scenario. If that was confirmed we could adopt an already accepted approach, instead of defining a new one. Indeed, this second case would have required an ad-hoc experimental campaign in our context, the publication in a journal and the consequent evaluation and possible adoption by research groups all over the world, in order to find possible weaknesses that could have been overlooked during our campaign.

In order to evaluate the existing classification algorithms, we decided to apply the classical measures adopted in the literature, i.e., Accuracy, Sensitivity and Specificity. If we indicate by: *(i)* *TP* the number of true positives, *(ii)* *TN* the number of true negatives, *(iii)* *FP* the number of false positives, and *(iv)FN* the number of false negatives, these three measures can be defined as:

$$ Accuracy = \frac{TP + TN}{TP + TN + FP + FN}$$

$$ Sensitivity = \frac{TP}{TP + FN}$$

$$ Specificity = \frac{TN}{TN + FP}$$

Accuracy is the number of correct forecasts on the total input size, and represents the overall performance of the algorithm. Sensitivity denotes the fraction of positive samples that are correctly identified. In our scenario, it is the fraction of *Fall Activities* that are properly identified by the algorithms. Finally, Specificity is the fraction of negative samples correctly identified, so it represents the fraction of *Not Fall Activities* properly identified by the algorithms. Accuracy, Sensitivity and Specificity are expressed by a number within the real interval [0,1]; the higher the value, the better the algorithm. However, in principle, it may happen that a high accuracy is achieved with an unacceptable time performance. Therefore, in addition to accuracy, we have considered a measure of performance. For this purpose, we have chosen the worst case time complexity of Training and Prediction activities.

In Table 4, we report a summary of all the classification algorithms tested by us. In particular, we report Accuracy, Sensitivity and Specificity, obtained through a 10-Fold Cross Validation, on the left, and some measures of Performance (i.e., the worst case time complexity for Training and Prediction activities) on the right. Worst case time complexity is expressed in Big *O* notation, where *n* is the number of training samples, *p* is the number of features, *n*_*sv*_ is the number of support vectors, $n_{l_{i}}$ is the number of neurons of layer *i*, *m* is the minimum between *n* and *p*.

**Table 4:** Accuracy, Sensitivity, Specificity values achieved by several classification algorithms when applied to our dataset (at left) and Worst Case Time Complexity of Training and Prediction (at right)

	Accuracy	Sensitivity	Specificity	Worst Case Time	Worst Case Time
				Complexity of Training	Complexity of Prediction
Decision Tree - C4.5	0.9487	0.9391	0.9566	*O*(*n*^2^*p*)	*O*(*p*)
Decision Tree - CART	0.9128	0.8910	0.9223	*O*(*n*^2^*p*)	*O*(*p*)
Multilayer Perceptron	0.9270	0.8829	0.9363	$O(n_{l_{0}}^{2})$	$O(pn_{l_{1}} + n_{l_{1}} n_{l_{2}} + ... )$
k-Nearest Neighbors (k = 3)	0.8790	0.8747	0.9263	*O*(*knp*)	*O*(*np*)
Logistic Regression	0.7707	0.8599	0.7057	*O*(*np*)	*O*(*p*)
Linear Discriminant Analysis	0.7557	0.4956	0.9663	*O*(*npm* + *m*^3^)	*O*(*np* + *nm* + *pm*)
Gaussian Naive Bayes	0.7175	0.4947	0.8989	*O*(*np*)	*O*(*p*)
Support Vector Machine	0.7141	0.4103	0.9486	*O*(*n*^2^*p* + *n*^3^)	*O*(*n*_*sv*_*p*)

A metric can be more important than another one, depending on the application scenario. In ours, i.e., detecting falls in a work environment, Sensitivity is more relevant than Specificity. Indeed, a missed alarm (corresponding to a *Not Fall Activity* prediction of a *Fall Activity*) means no assistance to the worker. On the other hand, a false alarm can be confirmed as such by the worker interacting with the device.

Looking at Table 4, we can see that the Decision Tree - C4.5 is the Machine Learning model with the highest Sensitivity and the highest Accuracy. Also the Specificity of this model is excellent. Actually, the Linear Discriminant Analysis achieved a Specificity of 0.9663, higher than the one of the Decision Tree - C4.5. However, it obtained a very low value of Sensitivity and a low Accuracy.

As for the worst case time complexity, we can observe that, for the Training activity, there are important differences between the various approaches. For example, k-Nearest Neighbors and Logistic Regression have a better worst case time complexity than Decision Tree. However, the Training activity is performed only once, when the device is adopted; it might be repeated during the device life, but it is still very rare. Actually, the most important activity, in terms of worst case time complexity is Prediction, which occurs continuously. As for this activity, the various approaches have very similar performances and, in any case, Decision Trees shows the best one.

Given all the classification algorithms of Table 4 and the previous reasoning, we found that the best algorithm for our scenario was Decision Tree - C4.5. Furthermore, the performances were so good that we could adopt it for our case, without thinking a new ad-hoc classification algorithm, which performances would hardly be the same and would be affected by all the problems mentioned at the beginning of this section.

In addition to Decision Tree C4.5, we decided to implement two auxiliary algorithms in our Personal Device. These, using accelerometric and gyroscopic data, evaluate the intensity of the movement and the position of the device to confirm or not a fall detected through C4.5. In fact, when a potential fall is reported as a result of C4.5 processing, the Personal Device verifies its own intensity of movement and, through it, can determine whether the operator wearing it is moving. If this intensity is zero or very low, the operator is most likely on the ground, without the capability of moving. In this case, the Personal Device uses the 6DoF (i.e., Six Degree of Freedom), provided by the two sensors embedded in it, to evaluate the position of the operator. If this is compatible with a fall (i.e., it is a supine or prone position), it concludes that there has been a fall and triggers the alarm.

#### Hardware framework of our wearable device

**Fig. 5:**
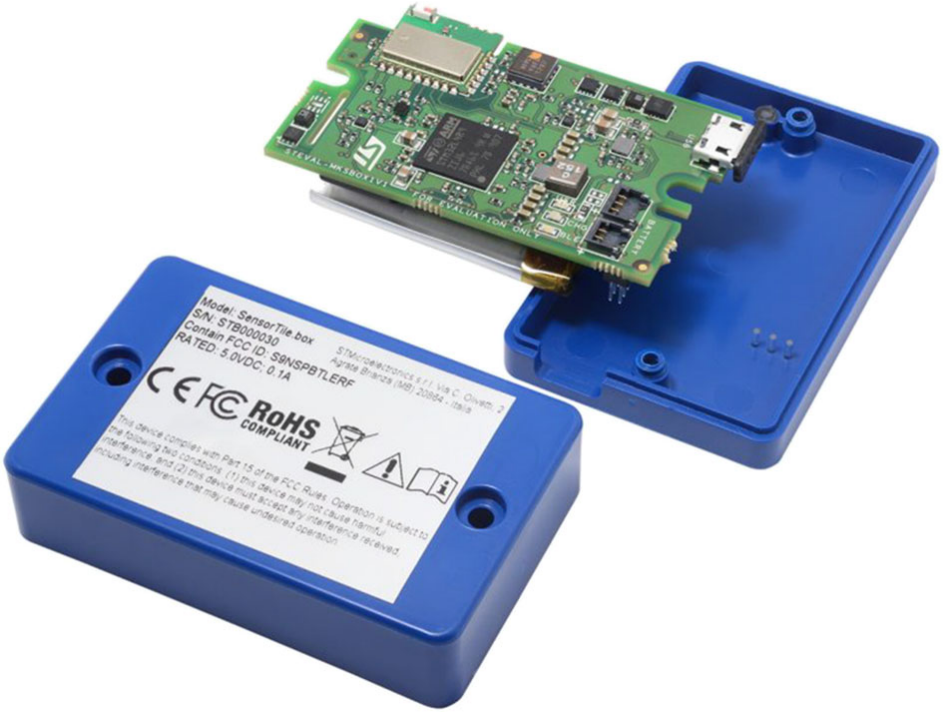
SensorTile.box (STEVAL-

Building the hardware framework of our device was difficult. Indeed, the device needed to implement our approach had to comply with some requirements. First, as said before, it had to be small and ergonomic, because it had to be worn by a person. Second, it should have an Inertial Measurement Unit (IMU), containing an accelerometer and a gyroscope. This was not sufficient because it also needed a Bluetooth module, able to manage the Bluetooth Low Energy (BLE) protocol. A device compliant with all these requirements is SensorTile.box provided by STMicroelectronics. It is shown in Fig. 5.

SensorTile.box was designed to support the development of wearable IoT devices. It has a BLE v4.2 module and an ultra-low-power microcontroller STM32L4R9 that manages the following sensors:

- STTS751, a high precision temperature sensor;
- LSM6DSOX, a six-axis IMU and Machine Learning Core (MLC);
- LIS3DHH and LIS2DW12, three-axis accelerometers;
- LIS2MDL, a magnetometer;
- LPS22HH, a pressure sensor;
- MP23ABS1, an analogic microphone;
- HTS221, a humidity sensor.

In the current version of our device, the only sensor we used is LSM6DSOX. However, we do not exclude that we will employ one or more of the other sensors in the future.

**Fig. 6:**
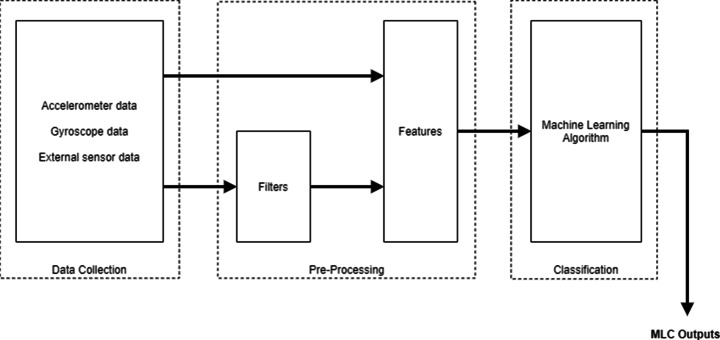
Workflow of the Machine Learning Core of LSM6DSOX

LSM6DSOX has everything our approach needs; it is a system-in-package (SIP) that contains a three-axis high precision accelerometer and a gyroscope. The really important feature of this sensor is the Machine Learning Core (MLC) component, in addition to its low power consumption and its small size. Thanks to this, we are able to implement Artificial Intelligence algorithms directly in the sensor, without the need for a processor. MLC exploits the data provided by the accelerometer, the gyroscope and some possible external sensors to compute some statistical parameters (such as mean, variance, maximum and minimum values, etc.) in a specific sliding time window. All these parameters can be the input of a classification algorithm (the decision tree in our case) previously loaded by the user. Fig. 6 reports the whole workflow of MLC.

In this figure, we specify some filters that can be applied to provided data. Examples of them are a low-pass filter, a bandwidth filter, a First-Order IIR and a Second-Order IIR. This feature was very important for our approach because it lets us implement the Butterworth filter, applied on the data provided by the accelerometer and the gyroscope to reduce noise (see Section 4.1.1).

#### Embedding the defined logics in our device

In order to embed the defined logics in our device, we had to develop a firmware accepted by SensorTile.box. It had to be written in the C language, with all the instructions needed for the initialization of the micro-controller and the configuration of the Machine Learning Core. In order to do this, STMicroelectronics provides two software tools (i.e., STM32CubeMX^5^ and STM32CubeIDE^6^) allowing users to develop C code for the microcontroller STM32L4R9. STM32CubeMX is a graphic tool to initialize the microcontroller peripherals, such as GPIO and USART, as well as its middlewares, like USB or TCP/IP protocols. The second software is an IDE allowing users to write, debug and compile the firmware of the microcontroller.

Our firmware has three essential functions, namely:

- HAL_init(), which starts the Hardware Abstraction Layer; this is a set of APIs above the hardware allowing the developer to interact with the hardware components safely.
- Bluetooth_init(), which initializes the Bluetooth stack. This operation includes the setting of the MAC address, the configuration of the HCI interface, the GAP and GATT protocols, and so forth.
- MLC_init(), which initializes the MLC component of LSM6DOX and enables the interruption of the output of decision trees. The MLC initialization is performed through the loading of a specific header file that configures all the registers of LSM6DOX. We describe this file below.

Configuring the MLC is not easy, because it also involves the configuration of the LSM6DSOX sensors and the settings of all its registers. This task is possible thanks to “Unico”^7^, a cross-platform Graphical User Interface developed by STMicroelectronics. Unico allows a quick and easy setup of the sensors, as well as the complete configuration of all the registers. It also provides the user with advanced features (such as the Machine Learning Core, a Finite State Machine, a pedometer, etc.) embedded in the digital output devices.

Thanks to Unico it is possible to configure all the parameters of MLC and LSM6DSOX sensors, like the output frequency of MLC, the full scale of the accelerometer and gyroscope, the sample window of reference for the computation of features, and so on. Our complete configuration is shown in Table 5.

**Table 5:** Adopted configuration of the MLC component

	Setting
Input data	Three axis accelerometer and gyroscope
MLC output frequency	12.5 Hz
Accelerometer sampling frequency	12.5 Hz
Gyroscope sampling frequency	12.5 Hz
Full scale accelerometer	± 8 g
Full scale gyroscope	± 2000 dps
Sample window	37 samples
Filtering	Second-Order IIR filter with cutting frequency at 4 Hz

With these settings, the output of the classification algorithm is written into a dedicated memory register at each clock of MLC, so it is possible to read the result. If this last is set to *Fall* (i.e., the worker wearing it has presumably fallen) the alarm is activated.

**Fig. 7:**
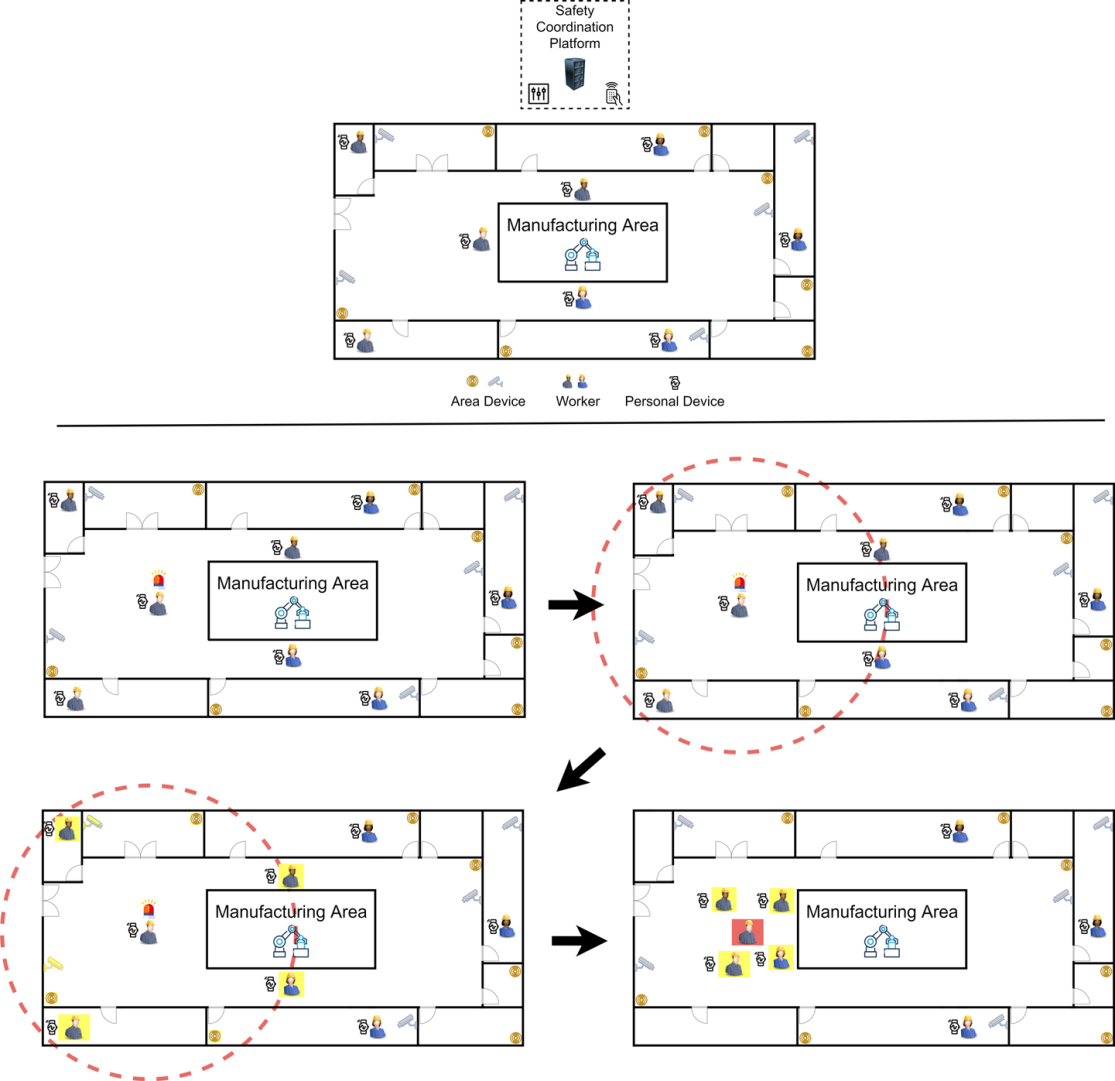
Example of a workplace scenario (on the top) and description of how the verification of a fall and the transmission of the alarms occur (on the bottom)

Figure 7 shows a possible workplace scenario, on the top, and how the verification of a fall and the transmission of the corresponding alarm occur, on the bottom. More specifically, each device continuously checks its status in order to trigger the alarm when needed. Whenever the MLC component of the device worn by a user detects a fall, it sends a broadcast alarm message. All the other Personal and Area Devices in the signal range receive the alarm. If there are Personal Devices in the same area, the corresponding workers are alerted to go to see what happened. In any case, the alarm reaches the Area Device that alerts the Safety Coordination Platform. This last examines the received data and, if the fall is confirmed, triggers the alarm, activates a rescue plan and sends the suitable advices to all the people involved in this plan.

As said before, all communications of our wearable device are carried out through the Bluetooth Low Energy (BLE) protocol. Our device has two roles, Central and Peripheral. The BLE protocol allows our device to switch from a role to another at runtime. When the device is running normal, it listens to any other device; this role is the Central one. When the worker falls and the MLC component detects this fall, the device switches to the Peripheral role, triggering the alarm activation and sending all the corresponding data.

#### Device testing

Once the logic of our approach was deployed in the SensorTile.box, we started the testing phase of our device. In particular, we asked 30 volunteers (15 males and 15 females of different age and weight) to perform different kinds of activities. The activities considered, reported in Table 6, include all the ones mentioned in the past literature. These could be grouped in *Fall Activities* and *Not Fall Activities*. Each time an activity was performed, the volunteer worn the device on the waist.

**Table 6:** A taxonomy for *Not Fall Activities* (on the left) and *Fall Activities* (on the right)

Not fall activity	Fall activity
Walk slow (< 6*km*/*h*)	Walk and fall forward after tripping
Walk fast (≥ 6*km*/*h*)	Walk and fall sideways (right) after tripping
Run slow (< 8*km*/*h*)	Walk and fall to the side (left) after tripping
Run fast (≥ 8*km*/*h*)	Fake fainting and fall on the right while standing
Sit slowly in a chair	Fake fainting and fall forward while standing
Sit slowly on the ground	Fake fainting and fall on the left while standing
Sit abruptly in a chair	Run and fall forward after stumbling
Jump to reach an object located at the top	
Go up and down the stairs slowly (< 6*km*/*h*)	
Go up and down the stairs quickly (≥ 6*km*/*h*)	
Walk and stumble without falling down	
Jump forward from an elevated position	
Jump forward from the floor	

Table 7 shows the confusion matrix obtained for the output provided by our device. Looking at this table, we can see that the real number of *Fall Activities* was 1,205. Our device correctly identified 1,170 of them, while 35 were false negatives. *Not Fall Activities* classified as such were 595. Our device recognized 540 of them, but triggered 55 false alarms. As we said before, Sensitivity is much more important than Specificity in this application context. Indeed, we obtained a higher number of real *Fall Activities* than the one of real *Not Fall Activities*. At the end, we have a Sensitivity value equal to 0.97; Specificity is equal to 0.91. Finally, Accuracy is equal to 0.95.

**Table 7:** Confusion matrix for the output provided by our device

	(Real) Fall	(Real) Not Fall
(Predicted) Fall	1170 (TP)	55 (FP)
(Predicted) Not Fall	35 (FN)	540 (TN)

The training and testing phases of our device show very satisfying performances. In our opinion, the training dataset, which we built starting from some existing datasets, was fundamental to obtain such successful results, because we were able to construct a general model from heterogeneous activities. Indeed, our model can distinguish between sport activities and falls, a difficult goal to achieve. Sensitivity is very high and that is the most important parameter to evaluate in our context. Specificity is not particularly high, which can lead to some false alarms. In most cases, these can be directly stopped by the other two auxiliary algorithms embedded in our device (see Section 4.1.3) or, ultimately, by the worker wearing the device. On the other hand, considering that our reference scenario is a working environment, activities like running or jumping are common. These could lead to many false alarms if the model would not be sufficiently generalized to handle them, fully or partially.

### Area Devices for fall detection

Area Devices represent the second level of our fall detection framework. They aim at monitoring a certain area of the working environment to check if one or more operators have fallen into it. In Section 2, we have seen that there are three types of fall detectors, i.e., ambient, video and wearable detectors. While wearable detectors belong to the Personal Devices category seen in the previous section, ambient and video detectors correspond to the Area Devices category.

An example of a fall detection approach using ambient detectors is proposed in [62]. It is based on the exploitation of a far-field microphone to record the audio of a given zone and classify possible falls. Instead, an example of a fall detection approach using video detectors is shown in [46]. It employs a single-camera system to detect a very large motion with a direction less than 180^∘^. However, these are only two of the many approaches that can be adopted for fall detection at the Area Devices level.

The big advantage of this kind of fall detector is the ability to evaluate several people in the same area at the same time, unlike Personal Devices that are able to evaluate only the person wearing them. Actually, Personal Devices and Area Devices can be leveraged as mutual validators. In fact, as we will see in the next section, Area Devices receive data about falls from Personal Devices and, by cross-referencing this data with the ones detected by them, they are able to support the Safety Coordination Platform of our framework in the detection of possible falls, in the activation of alarms, and in the management of rescue activities. In case a Personal Device and the corresponding Area Devices are in conflict (for example because the Personal Device reports a fall while an Area Device does not recognize it), our framework always chooses the most pessimistic case (i.e., it reports a fall). This is justified by the fact that, in our reference scenario, a false alarm is much more tolerable than a missed one. An alarm notification activates the procedure described in Fig. 7. If the alarm is triggered the operator is rescued; in the opposite case, the presence of a false alarm is reported.

### Safety Coordination Platform for fall detection

The Safety Coordination Platform monitors the working environment and, in case of operator falls, raises an alarm and coordinates rescue activities. A fundamental tool within the Safety Coordination Platform is a map that represents the working environment divided into its areas. The map shows all the Area Devices that, communicating directly with the platform, send useful data for monitoring falls. These data are retrieved from the sensors inside the Area Devices and from the Personal Devices that communicate them to the platform through the Area Devices. Once data arrive to the platform, the latter proceeds with several elaborations on it to extract knowledge about the presence or absence of falls. In case of a possible fall, the platform triggers the alarms and the corresponding rescue operations, taking into account the cause(s) that provoked the fall and the gravity of the latter.

**Fig. 8:**
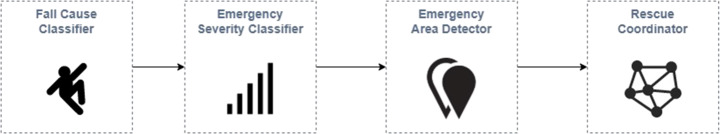
Modules of the safety coordination platform

The Safety Coordination Platform consists of a chain of four modules (Fig. 8):

- The *Fall Cause Classifier* aims at identifying the causes leading to a fall (e.g., slipping, fainting due to gas leaks, rushed escape due to fire or earthquake, etc.). For this purpose, it receives data from Area Devices and Personal Devices (through Area Devices) and passes it as input to one or more classification algorithms already proposed in past literature (e.g., Decision Tree, Support Vector Machine, Neural Networks, etc. [32]).
- The *Emergency Severity Classifier* examines the available data to identify the severity level of the emergency. This level is an integer between 1 and 5; the lower the value, the lower the severity is. The severity of an emergency depends on the type of a fall (for example, a slip is potentially less severe than a fall from the third floor), the cause of the fall (for example, a slip is potentially less severe than a fire) and the number of people involved. Also this classifier applies the data received from Area Devices to one or more classification algorithms already proposed in past literature.
- The *Emergency Area Detector* examines the available data to identify the area(s) involved in the emergency. For this purpose, it activates a clustering algorithm that groups the Area Devices and Personal Devices into those directly involved in the emergency, those indirectly involved in it (because, for example, they are involved in rescue activities), and those not involved in any way. The clustering algorithm determines the clusters taking into account the information related to the location of the various devices, as well as the type and level of the emergency (previously determined by the Fall Cause Classifier and the Emergency Severity Classifier).
- The *Rescue Coordinator* receives information on the cause of the fall, the severity of the emergency and the areas involved and, based on this information, it triggers the appropriate alarms. Next, it defines a rescue management plan (which may involve a rapid evacuation, a controlled evacuation, a simple first aid linked to a broken leg, etc.), providing each rescuer with the appropriate instructions. These are sent to the Area Devices, which, in turn, send, in broadcast mode, the advices to all the operators, who are working in the area (for example, requiring the immediate evacuation of the area, in case of gas leakage). Furthermore, Area Devices send, in broadcast mode, the advices for each Personal Device to be transmitted to the corresponding operator (for example, specifying the fastest way for her/him to reach the injured colleague to give first aid). Each Personal Device, thanks to the use of the appropriate actuators, provides the worker who wears it with the appropriate instructions on what to do and how doing it.

## Conclusion

In this paper, we proposed a new framework based on Sentient Multimedia Systems and Machine Learning to improve safety at work. First, we provided a general overview of the proposed framework. Then, we presented a more detailed description of its three layers, namely Personal Devices, Area Devices and Safety Coordination Platform. After that, in order to give a very concrete idea of how our framework can operate in reality, we illustrated its specialization to a typical scenario of safety at work, which is fall detection. With regard to this scenario, we described how our framework can be adopted to detect falls, activate alarms and coordinate rescue operations. In this description, we paid particular attention to Personal Devices as we introduced a new wearable device based on Machine Learning for fall detection in a workplace, which we designed, built and tested. Then, we took a look at Area Devices. Finally, we saw how the Safety Coordination Platform can operate to identify a fall, establish its cause and severity and, based on this information, define how to trigger alarms and how to organize and activate a rescue management plan.

As for future developments, we are planning to extend our work in several directions. First of all, we think of investigating metrics to evaluate Quality of Service (QoS) and Quality of Experience (QoE) from the worker perspective. Indeed, a continuous feedback from the users on the services they are employing and how they feel while working with our framework can help to identify some adjustments allowing an improvement in QoS and QoE.

Another interesting future development concerns the anonymization of data. In fact, in scenarios like those described in this paper, workers are surrounded by smart objects. These are certainly useful to increase their safety but, on the other hand, they are able to store a lot of data about workers that, properly combined, could allow the extraction of sensitive information about them. In order to address this problem, some popular database anonymization techniques, such as *k*-anonymity, *l*-diversity and *t*-closeness, could be included in our framework, to ensure that no information can be traced back to the specific worker, unless it is not required.

Finally, it is also interesting including in our framework smart objects able to evaluate the biometric parameters of the worker. Indeed, these could be fundamental to improve the prediction of negative events, such as falls, and to evaluate the level of stress of the worker during her/his activity. In fact, all the actions leading to an excessive level of stress are to be considered at risk, as they could lead the worker to a drop in concentration that could have disastrous effects on safety.

## Footnotes

1 https://ec.europa.eu/eurostat/statistics-explained/index.php/Accidents_at_work_statistics

2 Apache Kafka (https://kafka.apache.org/) is an open source distributed event streaming platform used for high-performance data pipelines, streaming analytics, data integration and mission-critical applications.

3 Kylo (https://www.kylo.io/) is an open source data lake management platform for self-service data ingestion and data preparation with integrated metadata management.

4 ELK stack (https://www.elastic.co/what-is/elk-stack) comprises three open source projects: *(i)* Elasticsearch, which is a search and analytics engine; *(ii)* Logstash, which is a server-side data processing pipeline that ingests data from multiple sources, transforms it and sends it to Elasticsearch; *(iii)* Kibana, which lets users visualize data with charts and graphs in Elasticsearch.

5 https://www.st.com/en/development-tools/stm32cubemx.html

6 https://www.st.com/en/development-tools/stm32cubeide.html

7 https://www.st.com/en/embedded-software/unico-gui.html
